# Identification of new biophysical markers for pathological ventricular remodelling in tachycardia‐induced dilated cardiomyopathy

**DOI:** 10.1111/jcmm.13699

**Published:** 2018-06-19

**Authors:** Aleyda Benitez‐Amaro, Valerie Samouillan, Esther Jorge, Jany Dandurand, Laura Nasarre, David de Gonzalo‐Calvo, Olga Bornachea, Gerard Amoros‐Figueras, Colette Lacabanne, David Vilades, Ruben Leta, Francesc Carreras, Alberto Gallardo, Enrique Lerma, Juan Cinca, Jose M. Guerra, Vicenta Llorente‐Cortés

**Affiliations:** ^1^ Group of Lipids and Cardiovascular Pathology ICCC Program Biomedical Research Institute Sant Pau (IIB Sant Pau) Hospital de la Santa Creu i Sant Pau Barcelona Spain; ^2^ Institute of Biomedical Research of Barcelona (IIBB) Spanish National Research Council (CSIC) Barcelona Spain; ^3^ CIRIMAT Université de Toulouse Université Paul Sabatier, Physique des Polymères Toulouse France; ^4^ CIBERCV Barcelona Spain; ^5^ Department of Cardiology Hospital de la Santa Creu i Sant Pau Biomedical Research Institute Sant Pau (IIB Sant Pau) Universitat Autonoma de Barcelona Barcelona Spain; ^6^ Department of Pathology Hospital de la Santa Creu i Sant Pau Barcelona Spain

**Keywords:** biophysical markers, cardiac remodelling, collagen, differential scanning calorimetry, fourier transform infrared spectroscopy, heart failure, myofiber

## Abstract

Our aim was to identify biophysical biomarkers of ventricular remodelling in tachycardia‐induced dilated cardiomyopathy (DCM). Our study includes healthy controls (N = 7) and DCM pigs (N = 10). Molecular analysis showed global myocardial metabolic abnormalities, some of them related to myocardial hibernation in failing hearts, supporting the translationality of our model to study cardiac remodelling in dilated cardiomyopathy. Histological analysis showed unorganized and agglomerated collagen accumulation in the dilated ventricles and a higher percentage of fibrosis in the right (RV) than in the left (LV) ventricle (*P *=* *.016). The Fourier Transform Infrared Spectroscopy (FTIR) 1st and 2nd indicators, which are markers of the myofiber/collagen ratio, were reduced in dilated hearts, with the 1st indicator reduced by 45% and 53% in the RV and LV, respectively, and the 2nd indicator reduced by 25% in the RV. The 3rd FTIR indicator, a marker of the carbohydrate/lipid ratio, was up‐regulated in the right and left dilated ventricles but to a greater extent in the RV (2.60‐fold vs 1.61‐fold, *P *=* *.049). Differential scanning calorimetry (DSC) showed a depression of the freezable water melting point in DCM ventricles – indicating structural changes in the tissue architecture – and lower protein stability. Our results suggest that the 1st, 2nd and 3rd FTIR indicators are useful markers of cardiac remodelling. Moreover, the 2nd and 3rd FITR indicators, which are altered to a greater extent in the right ventricle, are associated with greater fibrosis.

## 1 INTRODUCTION

Non‐ischemic dilated cardiomyopathy (DCM) is characterized by left ventricular (LV) dilatation and global systolic dysfunction with normal coronary arteries.^1^, ^2^ Progressive heart failure, ventricular and supraventricular arrhythmias, thromboembolisms and sudden death are the main clinical manifestations.^2^, ^3^, ^4^ In addition, DCM constitutes the most common cause of heart failure referred for cardiac transplantation.^5^ The ventricular remodelling resulting in ventricular dilatation and dysfunction has been extensively studied in vivo by means of non‐invasive techniques and post‐mortem in human and animal studies by histopathological and biochemical analysis.^6^, ^7^, ^8^, ^9^ However, the current knowledge of the mechanisms involved in the genesis of DCM is still limited. As a result, the treatments are scarce and have incomplete efficacy.

The molecular, conformational and physical characterization of the myocardium has emerged as a novel approach to study remodelling in diseased tissues. Spectrophotometric techniques, such as Fourier Transform Infrared Spectroscopy (FTIR) are powerful techniques that have been successfully applied to characterize cardiac and vascular tissues.^6^, ^7^, ^8^, ^9^, ^10^ Differential scanning calorimetry (DSC) is another suitable technique for characterizing biological tissues at the mesoscale, evaluating freezable and unfreezable water 11 and assessing protein thermal stability and conformational changes in tissues.^12^ DSC is particularly appropriate for evaluating the thermal stability of collagen in its purified or aggregated form,^13^ directly in cardiovascular tissues^14^ or in biomaterials.^15^ DSC has been also successfully applied to characterize the protein components of muscle tissues, such as myosin and its subfragments, actin and sarcoplasmic proteins.^16^, ^17^, ^18^ Nevertheless, few DSC data are available on the whole myocardium tissue, and no calorimetric data exist on tachycardia‐induced DCM.

The objective of the current investigation was to identify molecular, conformational and biophysical alterations useful as biophysical markers of cardiac remodelling in non‐ischemic dilated cardiomyopathy (DCM).

## 2 MATERIAL AND METHODS

### 2.1 Generation of a pig model of tachycardia‐induced DCM and experimental procedure

This study includes seventeen female domestic swine (Landrace‐Large White cross) weighing 54 ± 3 kg: seven control healthy animals (control group) and ten animals with tachycardia‐induced NIDCM (DCM group). The study protocol was approved by the Animal Care and Use Committee of our Institution, in accordance with the regulation for animal treatment established by the Guide for the Care and Use of Laboratory Animals (Eighth edition, National Research Council, Washington DC, The National Academies Press 2010). Detailed information about the generation of the pig model has been included in the Material and Methods section of the Supporting information. An electrocardiogram (ECG) and an echocardiogram were obtained (Model iE33, Philips Healthcare, The Netherlands) 15 minutes after the programmable pulse generator device was switched off in the DCM group to obtain an accurate calculation of the parameters. The heart rhythm and QRS width complex in the ECG were calculated. In the echocardiogram, the LV ejection fraction (LVEF) and the end‐diastolic (ED) and end‐systolic (ES) LV dimensions were measured. The femoral vein and artery were cannulated, and two catheters (Millar Instruments, Inc., Houston, TX*,* USA) were placed into the right (RV) and the left (LV) ventricles for measurement of the LV and RV pressures. Subsequently, a mid‐thoracotomy was performed. Before death, a bolus of pentobarbital was administered. The heart was excised, and samples from the RV and LV were immediately frozen at −80°C for molecular, biochemical and biophysical characterization. For the histological analysis, the myocardial samples were fixed, cryopreserved in 30% sucrose in phosphate‐buffered saline, embedded in Tissue‐Tek O.C.T. (Sakura^®^), and snap‐frozen in liquid nitrogen‐cooled isopentane.

### 2.2 Human control samples

Human autopsy hearts (n = 3) were obtained at Department of Pathology, Hospital Santa Creu i Sant Pau from deceased patients who died of non‐cardiac causes. Hearts were weighed, measured and samples from RV and LV ventricles were excised and frozen at −80°C for lipid characterization. The project was approved by the local Ethics Committee of Hospital de la Santa Creu i Sant Pau, Barcelona, Spain, and conducted in accordance with the guidelines of the Declaration of Helsinki. All patients gave written informed consent that was obtained according to our institutional guidelines.

### 2.3 Immunohistochemical analysis

#### 2.3.1 Myocardial fibrosis

It was assessed at the molecular level [analysis of the collagen type I and type III mRNA expression and protein levels] and by histological Masson's trichromic staining. For the latter, interstitial collagen deposition was assessed as the percentage of blue staining of 5 myocardial areas of 10 different immunohistochemical sections per heart using the ImageJ software.

#### 2.3.2 Determination of cardiomyocyte number

The amount of cardiomyocytes was calculated as the sum of nuclei observed in 5 fields at 40× of 10 different immunohistochemical sections.

#### 2.3.3 Determination of cardiomyocyte size

Sections of both venticles were stained with haematoxylin/Eosin. All the slides were analysed using an Olympus VANOX AHBT3 microscope and were photodocumented using a Sony DXC‐S500 camera. Longitudinal and transversal diameter of the cardiomyocytes were measured in at least 5 microscopic uniformly distributed fields at 40× of 10 different immunohistochemical sections per heart.

#### 2.3.4 Determination of presence of macrophages

For macrophage staining, after deparaffinization, antigen retrieval was performed for 20 minutes in 10 mmol/L/HCl buffer (pH 0) at 95°C. Endogenous peroxidase was blocked by 10% H_2_O_2_ in metanol for 30 minutes and after that with PBS‐Tween 0.1%‐serum horse 5% for 30 minutes at RT. Sections were then incubated with Avidin/Biotin blocking solution (Vector Laboratories Inc., Burlingame, CA, USA) for 15 minutes each. After washing with phosphate buffered saline (PBS) twice, sections were incubated with a mouse monoclonal [MAC387] (Abcam, ab22506) 1:200 in PBS‐Tween 0.1%‐serum horse 1% overnight. MAC387 stained sections were incubated with biotinylated antimouse made in horse for 1 hours followed by an incubation using a Vectastain ABC Elite kit (Vector Laboratories Inc.) for 1 hours. Reaction sites were visualized using an ImmPACT™ DAB Peroxidase Substrate Kit (Roche).

### 2.4 Real‐time PCR

Gene expression analyses of collagen type I (Ss03373340_m1; Applied Biosystems, Foster City, CA) and collagen type III (Ss04323794_m1; Applied Biosystems, Foster City, CA), Hexokinase 2 (Hk‐2; Hs00606086_m1; Applied Biosystems, Foster City, CA), glycogen phosphorylase (Ss03377042_u1; Applied Biosystems, Foster City, CA), and glycogen Synthase 1 (Ss03376867_u1; Applied Biosystems, Foster City, CA) were performed at the mRNA level by semi‐quantitative real‐time reverse transcriptase‐polymerase chain reaction (RT‐PCR). 18S rRNA (Mm.PT.58.43894205, IDT Conda, Integrated DNA Technologies, Inc.) was used as the internal control gene for amplification.

### 2.5 Western blot analysis

The protein levels of collagen and HSP70 were determined by Western blot analysis. Equivalent amounts of total protein were electrophoresed under reducing conditions on SDS‐polyacrylamide gels. The samples were electrotransferred to nitrocellulose membranes, which were then saturated at room temperature for 1 hours in TTBS (20 mm Tris‐HCl, pH 7.5, 500 mm NaCl, 0.01% Tween 20, and 5% non‐fat milk). Western blot analyses were performed with specific monoclonal antibodies against collagen type III (Abcam, clone FH‐7A ab6310) and heat shock proteins 70 (HSP70; Abcam, ab47455) and their corresponding secondary antibodies (1:10,000 dilution; Dako). Equal protein loading in each lane was verified staining filters with Pounceau and also by incubating the blots with monoclonal antibodies against Troponin T (Thermo Scientific, clone 13‐11, cardiac isoform Ab‐1).

### 2.6 Myocardial lipid content

Myocardial‐pulverized tissue (7 mg) from the porcine RV and LV was weighed and homogenized in 0.1 mol/L NaOH. The protein content of the extracts was quantified by a Pierce BCA Protein Assay (Thermo Fisher Scientific, Waltham, MA) to normalize the lipid content. Lipid extraction was performed as previously described.^19^, ^20^, ^21^, ^22^ The lipid extract was concentrated by evaporating the organic solvent under a N_2_ stream to prevent lipid oxidation. The cholesteryl ester (CE), free cholesterol (FC), triglyceride (TG) and phospholipid (PL) contents in the lipid extract were analysed by thin layer chromatography (TLC). Lipid extracts were loaded on silica G‐25 plates (DC‐Fertigplatten SIL G‐25 UV_254_) for CE, FC and TG and on an HPTLC glass plates silica gel 60 matrix for PL. A mixture of FC, CE and TG or a mixture of L‐α‐phosphatidylcholine (PC), L‐α‐phosphatidylethanolamine (PE), sphingomyelin (SM) and cardiolipin (CL) was applied to each of these types of plates plate type, respectively, as standards. The spots corresponding to CE, TG, FC, PE, PC, SM and CL were quantified by densitometry using a ChemiDoc system and Quantity‐One software (Bio‐Rad, Hercules, CA).

### 2.7 Vibrational characterization

One portion (5 mg) of myocardial tissue was freeze‐dried and used for vibrational characterization. Fourier transform infrared spectroscopy/attenuated total reflectance (FTIR/ATR) spectra of the freeze‐dried tissues were acquired using a Nicolet 5700 FTIR instrument (Thermo Fisher Scientific, Waltham, MA) equipped with an ATR device with a KBr beam splitter and a MCT/B detector. The ATR accessory used was a Smart Orbit with a type IIA diamond crystal (refractive index 2.4). Samples were directly deposited on the entire active surface of the crystal. For each sample, 80 interferograms were recorded in the 4000‐450/cm region, co‐added and Fourier transformed to generate an average spectrum of the segmented heart part with a nominal resolution of 1/cm using Omnic 8.0 (Thermo Fisher Scientific, Waltham, MA). A single‐beam background spectrum was collected from the clean diamond crystal before each experiment, and this background was subtracted from the spectra. Spectra were then subjected to ATR and baseline corrections and normalized in the amide II region. These spectra were next used in calculation of integrated bands intensities and their ratios. To quantify these various components, the areas of the different absorption bands were computed from the individual spectrum of each tissue, and the appropriate ratio of areas was used according to the literature data.^23^, ^24^ Second derivatives and Fourier self‐deconvolution (using *k* = 1.7 and half‐width = 13.5/cm) were used to enhance the chemical information present in overlapping infrared absorption bands of spectra. All spectra processing was performed using Omnic 8.0. The spectra presented for each group were calculated by averaging the spectra of all samples within each group.

### 2.8 Differential scanning calorimetry

Calorimetric analyses were performed using a DSC Pyris calorimeter (Perkin Elmer, Waltham, MA). The calorimeter was calibrated using Hg and In as standards, resulting in a temperature accuracy of ±0.1°C and an enthalpy accuracy of ±0.2 J/g. Fresh samples, 5‐10 mg in weight, were set into hermetic aluminium pans and equilibrated at the initial temperature for 5 minutes before cooling to −100°C at 10°C/min. Then, the thermograms were recorded during the heating at 10°C/minutes until reaching 90°C. Once DSC measurements were performed, the pans were reweighted to check that they had been correctly sealed. A second series of experiments were performed on freeze‐dried samples; in this case, freeze‐dried segmented parts of myocardium tissues (2‐3 mg) were set into standard aluminium pans and equilibrated at 20°C before recording thermograms during heating at 10°C/minutes until reaching 200°C. A detailed description of the procedure to calculate the amount of total, freezable and unfreezable water in hydrated proteins and tissues has been included in the material and methods section of the Supporting information.

### 2.9 Statistical analysis

Continuous variables are shown as the mean ± standard deviation (SD). Variables were compared between the groups using Student′s *t*‐test for independent samples and one‐way ANOVA, followed by Tukey's post hoc test, for comparison between each subgroup. Differences were considered to be statistically significant when *P *<* *.05.

## 3 RESULTS

### 3.1 Phenotype of pacing‐induced heart failure in DCM pigs

**Figure 1:**
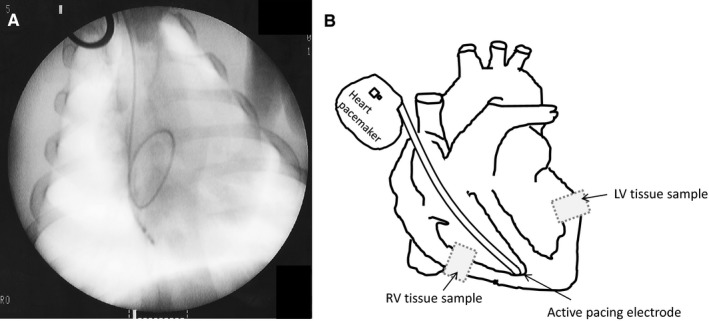
Pacing‐induced heart failure procedure in pigs. A, Fluoroscopy capture showing active pacing electrode fixed at the RV apex. B, Schematic heart representation depicting active pacing electrode position and LV and RV areas, where the tissue sample were collected. RV: right ventricle. LV: left ventricle

**Figure 2:**
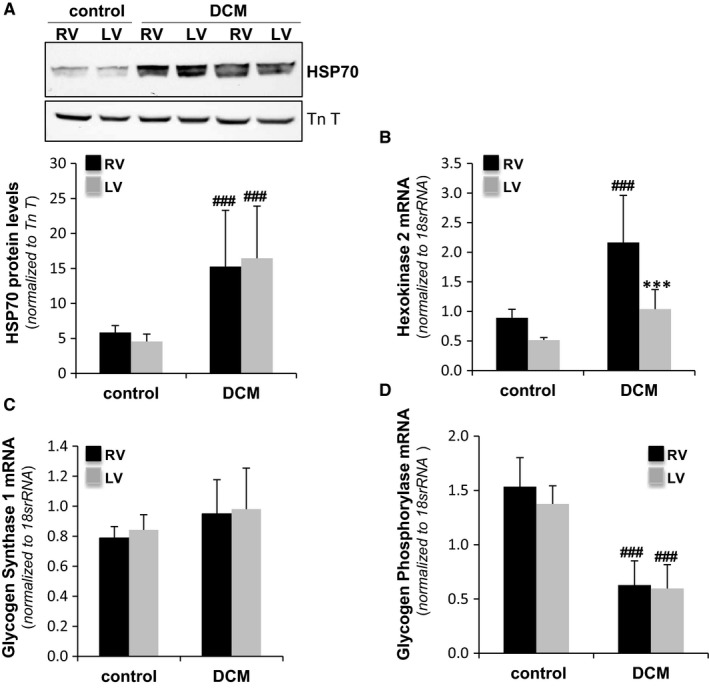
Molecular candidates of myocardial hibernation in the heart of DCM pigs. (A) Western blot showing the HSP70 band and bar graphs showing the quantification of HSP70 normalized to TnT, N = 7/group. Bar graphs showing the relative mRNA expression of hexokinase‐II (HK‐2) (B), glycogen synthase (GYSI) (C) and glycogen Phosphorylase (PYGM) (D) quantified by RT‐PCR. Values were normalized to *18S rRNA*, N = 7/group. ***P <* *.005 vs RV; ###*P *<* *.005 vs control. RV: right ventricle; LV: left ventricle

**Figure 3:**
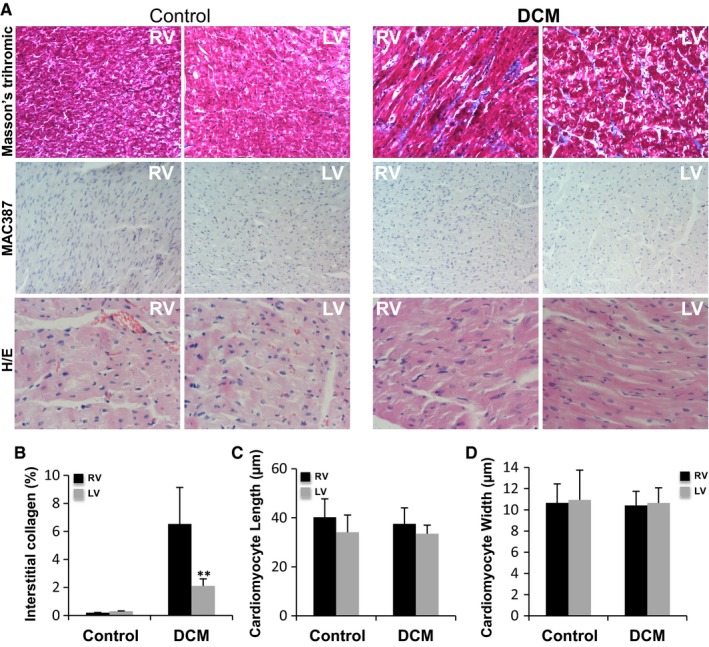
Histopathological and immunological characteristics of DCM hearts. (A) Masson's trichromic staining was used to distinguish cardiac muscle fibres (in red) and collagen (in green). Macrophage staining was performed incubating the sections with MAC387 monoclonal antibodies as explained in Methods. Haematoxylin/Eosin(H/E) staining was used to determine cardiomyocyte size. Cell nuclei were counterstained with DAPI (blue). Bar graphs showing fibrosis quantification (B), cardiomyocyte length (C) and cardiomyocyte width (D), N = 7/group. Results are expressed as the mean ± SD. **P <* *.01 vs RV. RV: right ventricle; LV: left ventricle

Animals in the DCM group underwent 23 ± 2 days of RV rapid pacing (detailed in Figure 1), and all developed dilated cardiomyopathy according to cardiac function parameters. As shown in Table 1, the left ventricle of these animals was dilated, and the ejection fraction was reduced. Furthermore, compared to the control group, DCM animals exhibited a wider QRS complex on the ECG (*P* < .001). The RV appeared to be subjectively dilated on echocardiography, and the dilatation was confirmed after the hearts were explanted. Haemodynamic measurements showed decreased contractility without a significant increase in the ventricular filling pressures. Previous studies have suggested that mechanical dyssynchrony, in the absence of coronary stenosis, can cause myocardial hibernation.^25^, ^26^, ^27^ Therefore, we tested the presence of molecular mechanisms associated to myocardial hibernation in our in vivo model. Western blot analysis showed that protein levels of HSP70, a protective heat shock protein, were significantly increased in both right and left ventricle of DMC compared to controls (Figure 2A), suggesting that this mechanism of cardiac protection is activated in DCM pigs. In addition, glycogen storage seems to be a key feature in the protection of hibernated myocytes.^28^, ^29^ Therefore, we measured cardiac gene expression of hexokinase 2 (HK‐2), glycogen phosphorylase (PYGM) and glycogen synthase (GYSI), enzymes involved in glycogen synthesis. Real‐time PCR results showed that HK‐2 was significantly increased in RV but not in LV of DCM pigs compared to controls (Figure 2B), GYSI was unaltered (Figure 2C), and PYGM was significantly down‐regulated in both ventricles (Figure 2D) of our in vivo model, suggesting an inactivation of glycogen degradation pathway in the heart of DCM pigs. Taken together, these results suggest that hybernation mechanisms are presented in both ventricles of DCM pigs. Masson's trichrome staining (Figure 3) showed extensive fibrosis in dilated ventricles consistent with the deposition of unorganized and agglomerated interstitial collagen. Quantification of the percentage of blue staining in immunohistochemical images revealed higher interstitial fibrosis in RV compared to LV of DCM pigs (Figure 3A and B). Haematoxilin/eosin staining allowed the measurement of cardiomyocyte amount, length and width. DCM pigs showed similar nuclei/area, length (Figure 3A and C) and width (Figure 3A and D) than control pigs, both in RV and LV. Comparing cardiomyocyte size from control pigs and humans, we have found that cardiomyocyte width is similar between pigs and humans, however, cardiomyocyte length in both RV and LV ventricles is lower in pigs than in humans (Figure S1). According to the scarce MAC387 staining, inflammation seems to be almost absent in porcine myocardial samples (Figure 3A).

**Table 1:** Electrocardiographic and echocardiographic parameters of the control and DCM groups

	Control	DCM	*P*
Electrocardiographic parameters			
Heart rate, bpm	80 ± 3	78 ± 6	ns
QRS width, ms	62 ± 2	97 ± 6	<.001
Echocardiographic parameters			
LVEF, %	72 ± 7	31 ± 10	<.001
LVEDD, mm	47 ± 5	59 ± 9	<.001
LVESD, mm	26 ± 4	48 ± 8	<.01
Haemodynamic parameters			
LV systolic pressure, mmHg	85 ± 5	73 ± 4	<.01
LV end diastolic pressure, mmHg	10 ± 2	11 ± 2	ns
LV dP/dt max, mmHg/s	1415 ± 173	557 ± 183	<.001
RV systolic pressure, mmHg	25 ± 7	28 ± 4	ns
RV end diastolic pressure, mmHg	3 ± 3	5 ± 3	ns
Central venous pressure, mmHg	5 ± 2	8 ± 2	.07
Pulmonary capillary pressure, mmHg	9 ± 2	9 ± 1	ns

Data are shown as the mean ± SEM. Control (N = 7), DCM (N = 10). LVEF, LV ejection fraction; LVEDD, LV end‐diastolic diameter; LVESD, LV end‐systolic diameter.

### 3.2 Pattern of FTIR bands in the myocardium of DCM pigs

**Figure 4:**
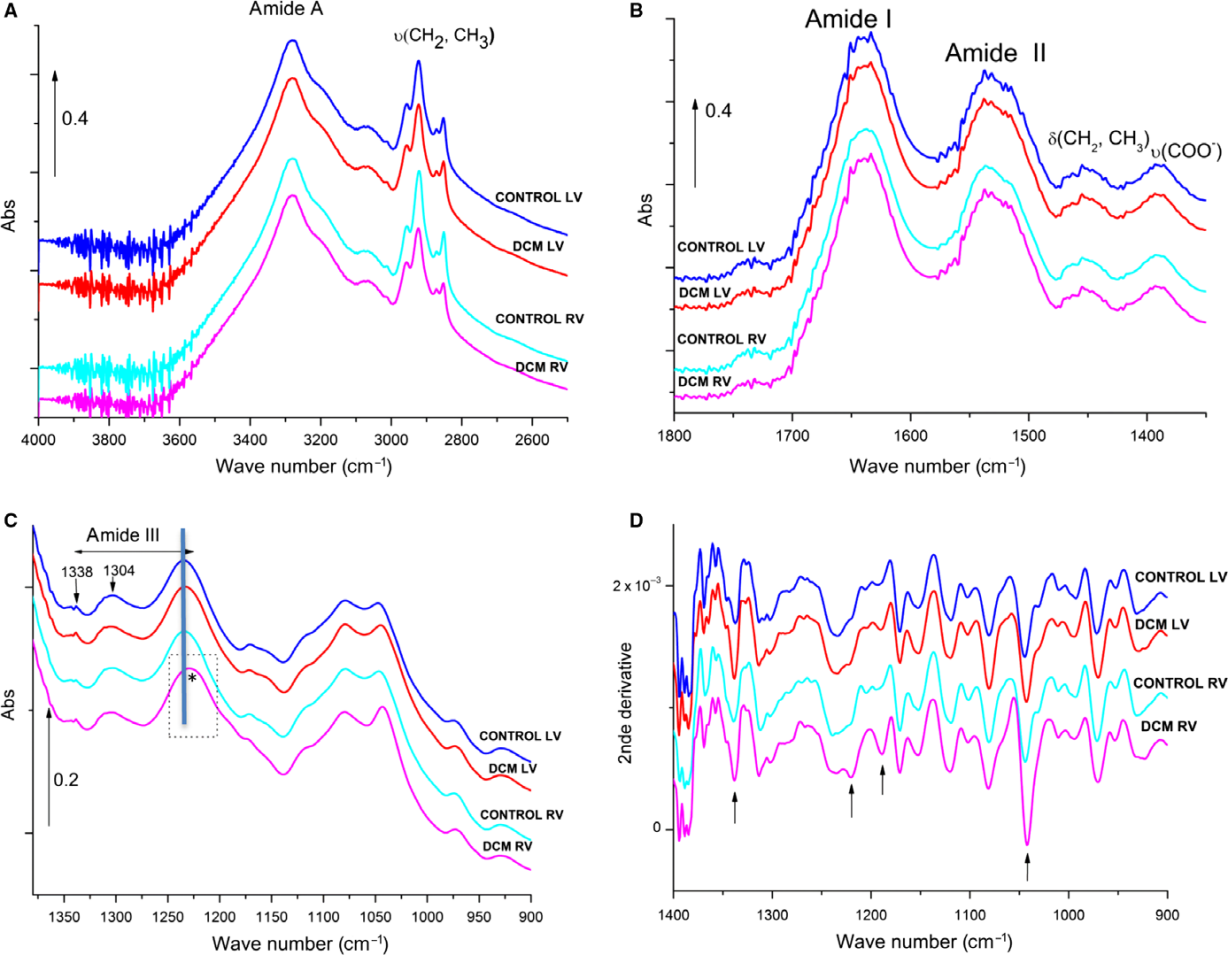
Averaged FTIR spectra of control and dilated ventricles. Line graph showing the 4000‐2500/cm (A), 1800‐1350/cm (B), 1350‐900/cm (C) spectra of control and dilated RV and LV. The asterisk indicates a shift of the absorption band in the dilated RV. Line graph showing the averaged second derivative spectra in the 1370‐900/cm region (D). Arrows indicate minima altered in dilated ventricles. N = 5/group. RV: right ventricle; LV: left ventricle

A detailed Table (Table S1) and description of the FTIR bands detected in porcine myocardial control tissue can be found in the Supporting Information. As shown in Figure 4A‐C, the major protein absorption bands reported in Table S1 for the control ventricles were conserved at the same wave numbers in the averaged spectra of the dilated ventricles, excepted the shift from 1234 to 1227/cm of the mixed absorption band in the amide III zone specifically in the dilated RV (Figure 4C, asterisk in the dotted box). There was also a smaller shift in LV, however, differently to that of RV, it did not reach statistical significance. The averaged second derivative spectra in the 1400‐900/cm zone showed an increase in the minima at 1338 (specific to collagen), 1220 (mainly proteoglycans), 1188 (mixed band, hardly assignable) and 1040 (collagens, carbohydrates residues and polysaccharides) per cm in the dilated ventricles (see arrows) (Figure 4D). The increase in the minima of these bands was more marked in the dilated RV. These results indicate molecular and conformational alterations in not only extracellular matrix proteins but also lipids and proteoglycans in dilated ventricles. The pattern of FTIR bands obtained in our in vivo DCM pig model indicates greater conformational remodelling associated with stronger alterations in the extracellular matrix proteins (collagen and proteoglycans) of the RV than of the LV.

### 3.3 FTIR indicators of myofibrillar and extracellular matrix proteins altered in DCM ventricles

**Figure 5:**
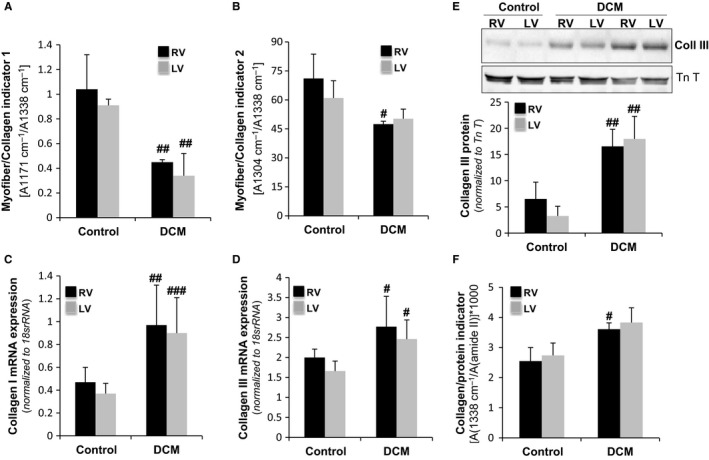
FTIR indicators of myofibrillar and extracellular matrix proteins are altered in DCM ventricles. Bar graphs showing FTIR myofiber/collagen indicator 1 (A) and myofiber/collagen indicator 2 (B), N = 5/group. Bar graphs showing the relative mRNA expression of collagen type I (C) and collagen type III (D) quantified by RT‐PCR. Values were normalized to *18S rRNA*, N = 7/group. (E) Western blot showing the collagen III band and bar graphs showing the quantification of collagen III normalized to TnT, N = 7/group. (F) Bar graphs showing the FITR collagen/protein indicator, N = 5/group. Results are expressed as the mean ± SD. #*P *<* *.05, ##*P *<* *.01, ###*P *<* *.005 vs control. RV: right ventricle; LV: left ventricle

Two distinct FTIR indicators of the myofiber/collagen ratio, the 1st [A(1171/cm)/A(1338/cm)] and 2nd [A(1304/cm (/A(1338/cm)] indicators (Figure 5A and B), revealed an imbalance between myofibers and collagen in dilated ventricles. FTIR 1st and 2nd indicators were reduced in dilated hearts, although the extent differed in each ventricle; the 1st indicator decreased by 45% in the RV and by 53% in the LV, and the 2nd indicator decreased by 25% in the RV. RT‐PCR and Western blot analysis showed a strong increase in collagen I mRNA (Figure 5C), collagen III mRNA (Figure 5D) and collagen III protein (Figure 5E) in dilated ventricles. Accordingly, the FTIR collagen/protein ratio indicator (Figure 5F) also showed a strong increase in the collagen fraction of dilated ventricles. Taken together, these results indicated exacerbated levels of collagen in dilated ventricles as a main cause of the imbalance between myofiber and collagen. In addition, the specific reduction of the 2nd indicator in the dilated RV suggested additional conformational alterations in this ventricle.

As shown in Figure S2B, the amounts of different protein secondary structures were mostly preserved in dilated ventricles. We only observed a slight increase in the band 1632/cm, which is assigned to β sheets structures, in the dilated samples.

### 3.4 Neutral lipid content (cholesteryl ester, free cholesterol, triglycerides, fatty acids and phospholipids) alterations in dilated ventricles

**Figure 6:**
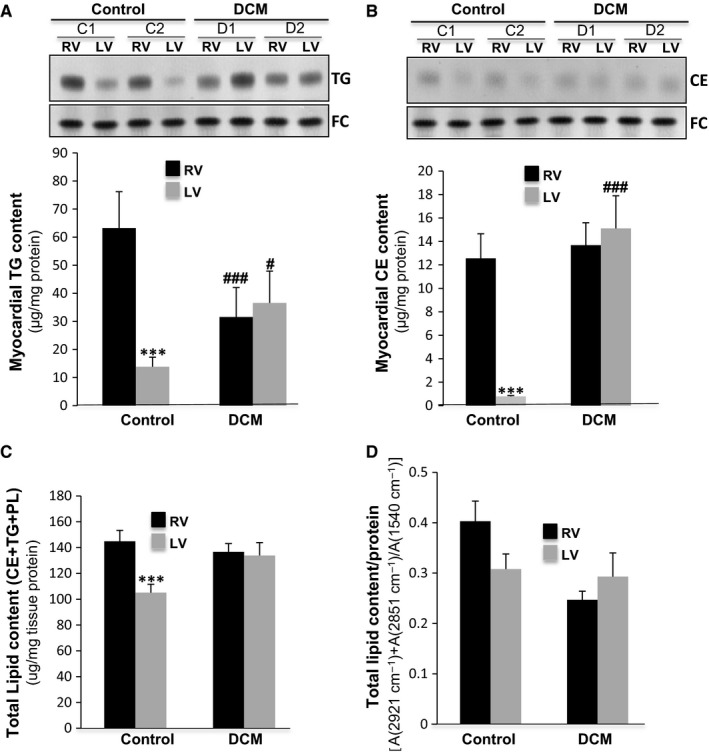
Neutral lipid content of ventricles is altered in DCM samples. Representative thin layer chromatography (TLC) and bar graphs showing the content of triglyceride (TG) (A) and cholesteryl esters (CE) (B) in control and DCM pigs. Total lipid (CE+TG+PL) content determined by biochemical (C) and FTIR (D), N = 7/group. Results are expressed as the mean ± SD. #*P *<* *.05, ###*P *<* *.005 vs control. ***P <* *.005 vs RV. RV: right ventricle; LV: left ventricle

Quantitative analysis of neutral lipids by thin layer chromatography (TLC) after lipid extraction showed that in control pigs, the RV contained much higher triglyceride (TG; Figure 6A) and cholesteryl ester (CE) levels (Figure 6B) than the LV. This strong difference in TG and CE between control RV and LV was reflected in the total neutral lipid content, as calculated from TLC data (*P* < .005; Figure 6C). There was also a clear tendency of neutral lipid content decrease in control LV measured by FITR indicators (Figure 6D), although in this case there were not significant differences at .05 level. To note, that differently to TLC data, FITR data included fatty acids. A detailed explanation of the FTIR bands assigned to lipids was provided in the Results section of the Supporting Information. The strong difference in myocardial TG and CE contents between the RV and LV in control was lost in the DCM group. This seems to be because of a decrease in the neutral lipid content in the RV concomitant with the increase in the LV in dilated cardiomyopathy (Figure 6A and B). Accordingly, there were no differences in the total lipid content measured by TLC (Figure 6C) or FTIR indicators (Figure 6D) between the dilated ventricles. The phospholipid pattern of the myocardium (described in detailed in the Result section of the Supporting Information) did not show differences between ventricles or groups (Figure S3).

### 3.5 FTIR indicators of proteoglycans and polysaccharides altered in DCM ventricles

**Figure 7:**
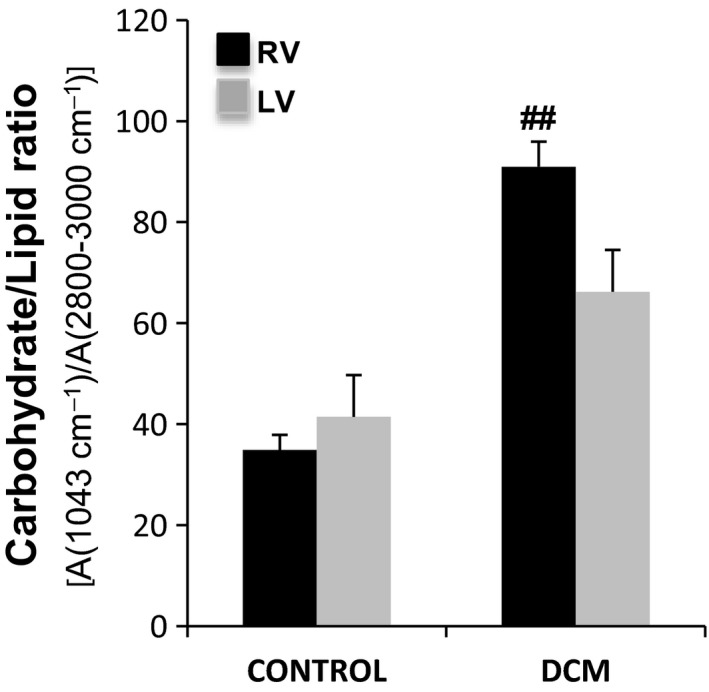
FTIR indicator of proteoglycans and polysaccharides. Carbohydrate/lipid ratio of the right and left ventricles in the control and dilated pigs, N = 5/group. Results are expressed as the mean ± SD. ##*P *<* *.01 vs control. RV: right ventricle; LV: left ventricle

Proteoglycans mainly contribute to the 1079/cm band and the specific band at 1226/cm, overlapping with protein amide III (Table S1). Glycogen and other polysaccharides contribute to the FTIR spectrum of the myocardium in the 1200‐1000/cm region (Figure 4C). The indicator A[(1043/cm)/A(2800‐3000/cm)], which is proportional to the carbohydrate/lipid ratio, was significantly increased in the right dilated ventricles (Figure 7). These results also suggest greater modifications in proteoglycans and polysaccharides in the right *versus* left dilated ventricle.

### 3.6 Thermal alterations in the myocardium of DCM pigs

**Figure 8:**
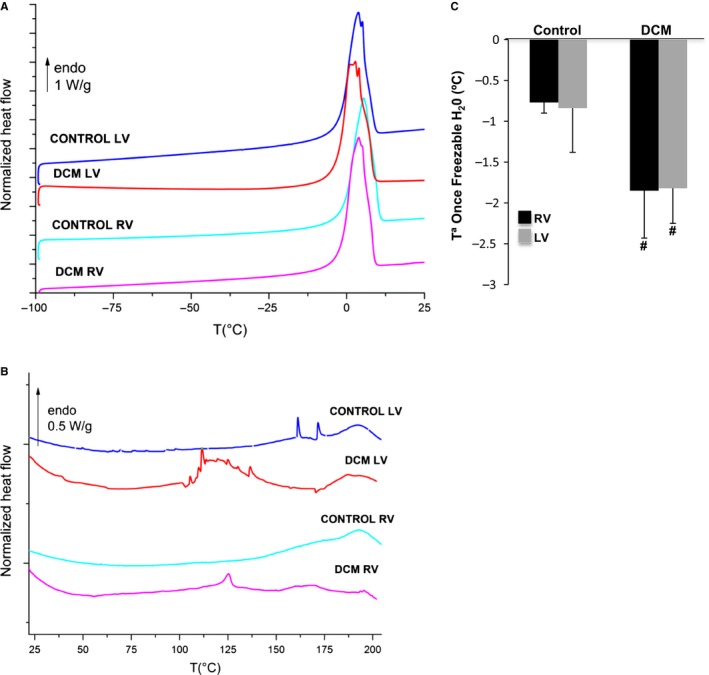
Thermal characterization of right and left ventricles in control and dilated pigs. A, DSC thermograms of fresh pig ventricles in the [−100; 25°C] region at 10°C/min. B, Onset melting temperature of freezable H_2_O.C, DSC thermograms of freeze‐dried pig ventricles in the [−20; 200°C] region at 10°C/min, N = 5/group. #*P *<* *.05 vs control

Figure 8A shows representative DSC thermograms (normalized to the initial mass) of fresh ventricles corresponding to the heating between −100 and 25°C. Thermograms were characterized by an endothermic peak in the [−10; 10°C] zone corresponding to the melting of previously frozen water. This peak is useful to calculate the amount of total, freezable and unfreezable water as explained in Material and Methods section of the Supporting Information. As shown in Figure S4, there were no differences in the total (Figure S4A), freezable (Figure S4B) or unfreezable H_2_O (Figure S4C) between the RV and LV of the control or DCM pigs. However, there was a significant depression of the onset of the ice‐melting temperature in dilated ventricles (Figure 8B). Figure 8C shows representative DSC thermograms (normalized to the initial mass) of freeze‐dried ventricles corresponding to the heating between 25 and 200°C. These thermograms were characterized by multiple endothermic events that correspond to the denaturation zone of cardiac muscle proteins. According to data in the DSC literature,^18^, ^30^ the major transitions characteristic of muscle are related to myosin, sarcoplasmic proteins, collagen and actin and occur in the [50‐85°C] range in the hydrated state. Although there are few DSC data available on freeze‐dried muscles, it is well‐known that the collagen denaturation occurring in the [60‐75°C] window in fresh sample is shifted towards the [180‐230°C] window in the freeze‐dried state because of the replacement of water–protein hydrogen bonds by protein–protein ones.^31^, ^32^ Therefore, the thermal transitions of other proteins of ventricles could follow a similar shift toward high temperature in the freeze‐drying process. In the current study, the thermal signature of the main proteins of control ventricles was localized in the [150‐200°C] zone, and it was largely altered in dilated ventricles. In particular, minor endothermic events were still found in the [150; 200°C] zone, but new and intense thermal phenomena were recorded in the [100; 150°C] zone. Taken together, our results indicate that the myocardial proteins in DCM pigs acquired reduced stability.

## 4 DISCUSSION

Our work identifies for the first time new biophysical markers of cardiac remodelling in non‐ischemic dilated cardiomyopathy. We have shown that the FTIR 1st and 2nd indicators (myofiber/collagen ratio), the FTIR 3rd indicator (carbohydrate/lipid ratio) and the DSC onset melting temperature of freezable water are biophysical markers of cardiac remodelling in dilated ventricles. Moreover, the FTIR 2nd and 3rd indicators, altered to a higher extent in right ventricle, are associated with greater fibrosis in a pig model of tachycardia‐induced cardiomyopathy.

First, we have validated our in vivo pig model as a translational model of dilated cardiomyopathy. It has been previously reported that heart failure is associated with global myocardial metabolism abnormalities typical of myocardial hibernation in different in vivo pig models.^33^, ^34^ Therefore, we tested several molecular candidates of myocardial hibernation in our in vivo model. We have measured protein levels of HSP70, heat shock proteins ubiquitously expressed that play a role in protein folding and exert protective effects. The high HSP70 levels that we have found in the dilated ventricles of our pig model indicate that this protective mechanism found in different in vivo models of heart failure also is present in our in vivo model. Previous studies also reported that glycogen storage is a key feature in the protection of hibernated myocytes.^28^, ^29^ Our results show that glycogen phosphorylase, mainly involved in the glycogen degradation pathway, was significantly down‐regulated in the both, RV but also in LV. These results indicate that, in our in vivo model, myocardial hybernation is not a restricted phenomena limited to the pacing site or pacing ventricle but a more extensive process affecting the whole heart, as previously described in other in vivo models of heart failure.^33^, ^34^ Like in these in vivo models, global myocardial metabolism alterations are compatible with differences in regional contractility. Indeed, we found crucial mechanical differences between right and left ventricles in our in vivo model. In addition, we have found other characteristics in the heart of our pigs, such as lower inflammatory state and minor variations in cardiomyocyte size that have been reported as associated to human DCM phenotype.^35^ Taken together, these results support the translationality and clinical utility of our in vivo model to analyse molecular and structural alterations associated with dilated cardiomyopathy. Our highly translational pig model showed dilatation in both cardiac chambers and decreased contractility with severe impairment of systolic function, in agreement with previous studies in in vivo models and patients.^36^, ^37^, ^38^, ^39^ In the current work, the physical parameters, in line with the immunohistochemical and molecular parameters, indicated strong cardiac remodelling of the extracellular matrix in dilated ventricles associated with the accumulation of collagen in an unorganized and agglomerated manner. The FTIR 1st and 2nd indicators suggest alterations in the myofiber/collagen ratio as a major factor contributing to collagen disorganization. Complementary information obtained by calorimetric parameters, particularly the onset melting temperature of freezable H_2_O, indicates a greater alteration in the tissue architecture of dilated ventricles. The shift of the denaturation zone towards low temperature in the freeze‐dried state marks the presence of newly synthesized collagen and proteins of lower thermal stability, which are susceptible to fragmentation or degradation. These results suggest that the reduction in the myofiber/collagen ratio underlying cardiac remodelling in DCM is caused not only by the increase in collagen but also by the presence of fragmented myofibrillar and extracellular matrix proteins.

Our model showed a high degree of fibrosis in both dilated ventricles, although the extent was greater in the right ventricle. The extended fibrosis in the RV is associated with a greater reduction of 2nd FTIR indicator (myofiber/collagen ratio) and a significant increase in FTIR 3rd indicator (carbohydrate/lipid ratio). The 3rd indicator augmentation seems to be related, at least in part, to the decrease in triglyceride (TG) content specifically in the right dilated ventricles. Our study revealed higher TG and CE contents in the RV than in the LV of control animals. This phenotype is consistent with the higher effort and energetic consumption needs of a healthy LV. In DCM pigs, these differences in the neutral lipid content between ventricles are lost because of decrease in the RV, concomitant with an increase in the LV. Different research groups including ours have reported an increased TG myocardial accumulation in several cardiomyopathies, including dilated,^19^ ischemic 20, 21, 22 and diabetic cardiomyopathy.^40^, ^41^, ^42^ In this particular pacing‐induced DCM model, the TG increase occurs in the LV, while the opposite occurs in the RV. This finding suggests important differences in the progression of the RV and LV from a healthy to a pathological state, at least in terms of lipid accumulation. In line with our results, previous studies have shown that tachycardia decreases the TG content of the RV in a rat model of dilated cardiomyopathy.^43^ The authors propose that pacing places a greater burden on the RV and that this justifies higher TG mobilization. Additionally, in accordance with our results, a previous study using a Syrian hamster model reported that cardiomyopathy progresses with an increase in ECM and a decrease in cellular lipids.^24^

In addition to the global myocardial metabolism abnormalities (typical of myocardial hibernation) and to the alterations in TG and CE content in the hearts of DCM pigs, we also observed a reduction in creatine levels (quantified by the specific band at 1304/cm,^44^ Table S1). Taken together, these results suggest that structural remodelling in this pig model is closely associated with metabolic derangements. These results support the translational nature of our pig model because, in humans, dilated cardiomyopathy occurs with a progressive reduction in creatin.^45^, ^46^ Moreover, a tight relation between alterations in creatin levels and the severity of heart failure estimated by ejection fraction has been previously reported in humans.^46^, ^47^

In conclusion, our work identifies the FTIR 1st and 2nd indicators (myofiber/collagen ratio), the FTIR 3rd indicator (carbohydrate/lipid ratio) and the DSC onset melting T^a^ of freezable H_2_O as biophysical markers of cardiac remodelling in dilated cardiomyopathy. The combination of vibrational and calorimetric data support that both accumulation of interstitial collagen and thermal instability of myofibers and ECM proteins contribute to the imbalance in the myofiber/collagen ratio and to the accumulation of unorganized and agglomerated collagen in dilated ventricles. Moreover, greater alterations in the FTIR 2nd and 3rd indicators are associated with higher fibrosis in the right dilated ventricles.

## 5 CLINICAL IMPACT

Our results, obtained in a high translational model using novel research techniques, provide new key biophysical markers of pathological ventricular remodelling that are useful for the characterization of dilated cardiomyopathy. Interestingly, these biophysical markers showed significant differences between the right and the left ventricles, indicating ventricle‐specific remodelling alterations. In addition, the study supports the concept that ultrastructural alterations persist in tachycardiomyopathy‐induced cardiomyopathy, as some clinical studies have suggested.

## CONFLICT OF INTEREST

None.

## References

[1] Maron BJ , Towbin JA , Thiene G , et al. Contemporary definitions and classification of the cardiomyopathies. Circulation. 2006;113:1807‐1816. 16567565 10.1161/CIRCULATIONAHA.106.174287

[2] Jefferies JL , Towbin JA . Dilated cardiomyopathy. Lancet (London, England). 2010;375:752‐762. 10.1016/S0140-6736(09)62023-7 20189027

[3] Towbin JA , Bowles NE . The failing heart. Nature. 2002;415:3. 10.1038/415227a 11805847

[4] Roura S , Bayes‐Genis A . Vascular dysfunction in idiopathic dilated cardiomyopathy. Nat Rev Cardiol. 2009;6:590‐598. 19636323 10.1038/nrcardio.2009.130

[5] Hershberger RE , Hedges DJ , Morales A . Dilated cardiomyopathy: the complexity of a diverse genetic architecture. Nat Rev Cardiol. 2013;10:531‐547. 23900355 10.1038/nrcardio.2013.105

[6] Bromberg PS , Gough KM , Dixon IM . Collagen remodeling in the extracellular matrix of the cardiomyopathic Syrian hamster heart as assessed by FTIR attenuated total reflectance spectroscopy. Can J Chem. 1999;77:1843‐1855.

[7] Gough KM , Zelinski D , Wiens R , et al. Fourier transform infrared evaluation of microscopic scarring in the cardiomyopathic heart: effect of chronic AT1 suppression. Anal Biochem. 2003;316:232‐242. 12711345 10.1016/s0003-2697(03)00039-3

[8] Liu KZ , Dixon IM , Mantsch HH . Distribution of collagen deposition in cardiomyopathic hamster hearts determined by infrared microscopy. Cardiovasc Pathol. 1999;8:41‐47. 10722247 10.1016/s1054-8807(98)00024-6

[9] Cheheltani R , Rosano JM , Wang B , et al. Fourier transform infrared spectroscopic imaging of cardiac tissue to detect collagen deposition after myocardial infarction. J Biomed Opt. 2012;17:56014. 10.1117/1.JBO.17.5.056014 PMC3381018 22612137

[10] Kuwahara M , Bannai K , Segawa H , et al. Cardiac remodeling associated with protein increase and lipid accumulation in early‐stage chronic kidney disease in rats. Biochim Biophys Acta. 2014;1842:1433‐1443. 24798235 10.1016/j.bbadis.2014.04.026

[11] Aktas N , Tülek Y , Gökalp HY . Determination of freezable water content of beef semimembranous muscle DSC study. J Therm Anal. 1997;48:259‐266.

[12] Samouillan V , Dandurand J , Lacabanne C , et al. Comparison of chemical treatments on the chain dynamics and thermal stability of bovine pericardium collagen. J Biomed Mater Res A. 2003;64:330‐338. 12522820 10.1002/jbm.a.10326

[13] Miles CA , Burjanadze TV , Bailey AJ . The kinetics of the thermal denaturation of collagen in unrestrained rat tail tendon determined by differential scanning calorimetry. J Mol Biol. 1995;245:437‐446. 7837274 10.1006/jmbi.1994.0035

[14] Samouillan V , Dandurand J , Lacabanne C , et al. Characterization of aneurysmal aortas by biochemical, thermal, and dielectric techniques. J Biomed Mater Res A. 2010;95:611‐619. 20725971 10.1002/jbm.a.32835

[15] Lee J , Pereira C , Abdulla D , et al. A multi‐sample for collagenous denaturation biomaterials temperature tester. Mol Eng Phys. 1995;17:115‐121. 10.1016/1350-4533(95)91882-h 7735640

[16] Wright DJ , Wilding P . Differential scanning calorimetric study of muscle and its proteins: myosin and its subfragments. J Sci Food Agric. 1984;35:357‐372. 6368978 10.1002/jsfa.2740350317

[17] Dergez T , Könczöl F , Farkas N , et al. DSC study of glycerol‐extracted muscle fibers in intermediate states of ATP hydrolysis. J Therm Anal Calorim. 2005;80:445‐449.

[18] Tamilmani P , Pandey MC . Thermal analysis of meat and meat products. J Therm Anal Calorim. 2016;123:1899‐1917.

[19] Roura S , Gálvez‐Montón C , de Gonzalo‐Calvo D , et al. Extracellular vesicles do not contribute to higher circulating levels of soluble LRP1 in idiopathic dilated cardiomyopathy. J Cell Mol Med. 2017;21:3000‐3009. 28557183 10.1111/jcmm.13211 PMC5661250

[20] Cal R , Castellano J , Revuelta‐López E , et al. Low‐density lipoprotein receptor‐related protein 1 mediates hypoxia‐induced very low density lipoprotein‐cholesteryl ester uptake and accumulation in cardiomyocytes. Cardiovasc Res. 2012;94:469‐479. 22454363 10.1093/cvr/cvs136

[21] Castellano J , Farré J , Fernandes J , et al. Hypoxia exacerbates Ca(2 + )‐handling disturbances induced by very low density lipoproteins (VLDL) in neonatal rat cardiomyocytes. J Mol Cell Cardiol. 2011;50:894‐902. 21338608 10.1016/j.yjmcc.2011.02.002

[22] Cal R , Juan‐Babot O , Brossa V , et al. Low density lipoprotein receptor‐related protein 1 expression correlates with cholesteryl ester accumulation in the myocardium of ischemic cardiomyopathy patients. J Transl Med. 2012;10:160. 22873206 10.1186/1479-5876-10-160 PMC3479056

[23] Staniszewska E , Malek K , Baranska M . Rapid approach to analyze biochemical variation in rat organs by ATR FTIR spectroscopy. Spectrochim Acta A Mol Biomol Spectrosc. 2014;118:981‐986. 24161861 10.1016/j.saa.2013.09.131

[24] Wang Q , Sanad W , Miller LM , et al. Infrared imaging of compositional changes in inflammatory cardiomyopathy. Vib Spectrosc. 2005;38:217‐222.

[25] Lionetti V , Aquaro GD , Simioniuc A , et al. Severe mechanical dyssynchrony causes regional hibernation‐like changes in pigs with nonischemic heart failure. J Card Fail. 2009;15:920‐928. 19944370 10.1016/j.cardfail.2009.06.436

[26] Nikolaidis LA , Hentosz T , Doverspike A , et al. Mechanisms whereby rapid RV pacing causes LV dysfunction: perfusion‐contraction matching and NO. Am J Physiol Heart Circ Physiol. 2001;281:H2270‐H2281. 11709392 10.1152/ajpheart.2001.281.6.H2270

[27] Lionetti V , Matteucci M , Ribezzo M , et al. Regional mapping of myocardial hibernation phenotype in idiopathic end‐stage dilated cardiomyopathy. J Cell Mol Med. 2014;18:396‐414. 24444256 10.1111/jcmm.12198 PMC3955147

[28] Kim S‐J , Peppas A , Hong S‐K , et al. Persistent stunning induces myocardial hibernation and protection: flow/function and metabolic mechanisms. Circ Res. 2003;92:1233‐1239. 12750311 10.1161/01.RES.0000076892.18394.B6

[29] Ofir M , Arad M , Porat E , et al. Increased glycogen stores due to gamma‐AMPK overexpression protects against ischemia and reperfusion damage. Biochem Pharmacol. 2008;75:1482‐1491. 18261713 10.1016/j.bcp.2007.12.011

[30] Schubring R . Characterizing protein changes caused by application of high hydrostatic pressure on muscle food by means of DSC. J Therm Anal Calorim. 2005;82:229‐237.

[31] Samouillan V , Delaunay F , Dandurand J , et al. The use of thermal techniques for the characterization and selection of natural biomaterials. J Funct Biomater. 2011;2:230‐248. 24956305 10.3390/jfb2030230 PMC4030942

[32] Miles CA , Ghelashvili M . Polymer‐in‐a‐box mechanism for the thermal stabilization of collagen molecules in fibers. Biophys J. 1999;76:3243‐3252. 10354449 10.1016/S0006-3495(99)77476-X PMC1300293

[33] Lionetti V , Guiducci L , Simioniuc A , et al. Mismatch between uniform increase in cardiac glucose uptake and regional contractile dysfunction in pacing‐induced heart failure. Am J Physiol Heart Circ Physiol. 2007;293:H2747‐H2756. 17704291 10.1152/ajpheart.00592.2007

[34] Czuriga D , Tóth A , Pásztor ET , et al. Cell‐to‐cell variability in troponin I phosphorylation in a porcine model of pacing‐induced heart failure. Basic Res Cardiol. 2012;107:244. 22237651 10.1007/s00395-012-0244-x PMC3329882

[35] Mueller KAL , Heinzmann D , Klingel K , et al. Histopathological and Immunological Characteristics of Tachycardia‐Induced Cardiomyopathy. J Am Coll Cardiol. 2017;69:2160‐2172. 28449778 10.1016/j.jacc.2017.02.049

[36] Patel HJ , Pilla JJ , Polidori DJ , et al. Ten weeks of rapid ventricular pacing creates a long‐term model of left ventricular dysfunction. J Thorac Cardiovasc Surg. 2000;119:834‐841. 10733777 10.1016/S0022-5223(00)70021-3

[37] Tanaka R , Spinale FG , Crawford FA , et al. Effect of chronic supraventricular tachycardia on left ventricular function and structure in newborn pigs. J Am Coll Cardiol. 1992;20:1650‐1660. 1452940 10.1016/0735-1097(92)90462-v

[38] Zellner JL , Spinale FG , Eble DM , et al. Alterations in myocyte shape and basement membrane attachment with tachycardia‐induced heart failure. Circ Res. 1991;69:590‐600. 1873861 10.1161/01.res.69.3.590

[39] Ohno M , Cheng CP , Little WC . Mechanism of altered patterns of left ventricular filling during the development of congestive heart failure. Circulation. 1994;89:2241‐2250. 8181149 10.1161/01.cir.89.5.2241

[40] Samouillan V , Revuelta‐López E , Dandurand J , et al. Cardiomyocyte intracellular cholesteryl ester accumulation promotes tropoelastin physical alteration and degradation: role of LRP1 and cathepsin S. Int J Biochem Cell Biol. 2014;55:209‐219. 25218173 10.1016/j.biocel.2014.09.005

[41] van der Meer RW , Rijzewijk LJ , Diamant M , et al. The ageing male heart: myocardial triglyceride content as independent predictor of diastolic function. Eur Heart J. 2008;29:1516‐1522. 18492680 10.1093/eurheartj/ehn207

[42] van der Meer RW , Hammer S , Smit JWA , et al. Short‐term caloric restriction induces accumulation of myocardial triglycerides and decreases left ventricular diastolic function in healthy subjects. Diabetes. 2007;56:2849‐2853. 17717279 10.2337/db07-0768

[43] Wojcik B , Harasim E , Zabielski P , et al. Effect of tachycardia on lipid metabolism and expression of fatty acid transporters in heart ventricles of the rat. J Physiol Pharmacol. 2015;66:691‐699. 26579575

[44] Jerônimo DP , de Souza RA , da Silva FF , et al. Detection of creatine in rat muscle by FTIR spectroscopy. Ann Biomed Eng. 2012;40:2069‐2077. 22419197 10.1007/s10439-012-0549-9

[45] Nascimben L , Ingwall JS , Pauletto P , et al. Creatine kinase system in failing and nonfailing human myocardium. Circulation. 1996;94:1894‐1901. 8873665 10.1161/01.cir.94.8.1894

[46] Neubauer S . The failing heart–an engine out of fuel. N Engl J Med. 2007;356:1140‐1151. 17360992 10.1056/NEJMra063052

[47] Nakae I , Mitsunami K , Yoshino T , et al. Clinical features of myocardial triglyceride in different types of cardiomyopathy assessed by proton magnetic resonance spectroscopy: comparison with myocardial creatine. J Card Fail. 2010;16:812‐822. 20932463 10.1016/j.cardfail.2010.05.006

