# Supplementary material for: Identification of new biophysical markers for pathological ventricular remodelling in tachycardia‐induced dilated cardiomyopathy
Source: J Cell Mol Med. 2018 Jun 19;22(9):4197–208. doi: 10.1111/jcmm.13699 (PMC6111813; doi:10.1111/jcmm.13699)
Supplement: Supplementary file 1 [file JCMM-22-4197-s001.docx]

**SUPPORTING INFORMATION**

**METHODS**

**Generation of the pig model of tachycardia-induced dilated cardiomyopathy (DCM)**

Four weeks prior to the experimental protocol, the animals of the DCM group were pre-medicated with a combination of ketamine (12 mg/kg) and midazolam (0.6 mg/kg) injected intramuscularly and anesthetized using propofol (2-4 mg/kg iv). After endotracheal intubation, general anaesthesia was maintained with a mixture of oxygen and sevoflurane (2.5-3.5%). Ventilatory parameters were adjusted to maintain blood gases within physiological ranges. Remifentanil (0.2 mcg/kg/min iv) was administered during the procedure for analgesia, and a fentanyl transdermal release patch was used to assure correct analgesia in the immediate postoperative period. A unipolar, active fixation endocardial pacing electrode (1888TC/58 cm, Sant Jude Medical Inc., USA) was advanced to the right ventricular apex under fluoroscopic guidance through the right internal jugular vein using sterile technique. The appropriate lead position was acceptable for R-wave sensing above 4 mV, impedance between 200-2000 Ω and pacing threshold of 2 V at 0.4 ms. The pacing lead was connected to a programmable pulse generator (Accent RF pacemaker, St Jude Medical Inc., USA), and the pacing system was placed in a subcutaneous pocket in the neck. The animals were allowed to recover for 1 week. The pacemaker was then programmed to a mode of constant ventricular pacing without sensing (VOO) at a rate of 200 beats/min and an output of 5.0 volts with a 0.4 ms pulse width for 3 weeks.

**Procedure to calculate the amount of total, freezable and unfreezable water in hydrated tissues**

Thermograms are characterized by an endothermic peak in the [-10; 10°C] zone or the transition corresponding to the melting of previously frozen water. This transition is widely used to quantify the amount of total freezable water in hydrated proteins and tissues (by dividing the area of the measured endothermic peak by 334 J.g^-1^, corresponding to the melting enthalpy of pure ice at 0°C) [1]. The sample pans were pierced and dried to constant weight at 195 °C for 10 minutes to determine the sample dry weight and the total water content. The amount of unfreezable water can be calculated by a simple difference.

**RESULTS**

**Myocardial FTIR protein pattern (myofibrillar proteins and sarcoplasmic and structural proteins of the extracellular matrix) in control pigs.**

Table S1 summarizes the FTIR bands detected in porcine control tissue (Figure 4A, 4B, and 4C) and their assignments according to previous published data [2–9] . The major absorptions in such spectra are amide A, e amide I and amide II, which are mainly associated with proteins in freeze-dried tissues. The main ventricular proteins by mass are cardiomyocyte myofibrillar proteins (myosin, α-actin), sarcoplasmic proteins, and the structural proteins of the extracellular matrix (ECM), mainly collagens. Among the five types of collagen (I, III, IV, V, VI) identified in the heart, the fibrillar collagens I and III (that colocalize) represent more than 90% of the total collagens. Previous studies showed that collagens (I, III, IV, V, and VI) exhibit a specific triplet at 1204, 1238, and 1280 cm^-1^ (arising from the amide III vibration) and a specific absorption at 1338 cm^-1^ (wagging of proline side chain) [2, 10, 11]. Moreover, comparison between the FTIR spectra of muscle and its separated components -myofibrils and extracellular matrix- corroborated the absence of these four mentioned absorption bands in myofibers [6] . Among these different vibrations, the 1338 cm^-1^ band is the specific signature of the ECM structural proteins since it is the only band that does not overlap with the absorption or other components, such as DNA, lipids or proteoglycans. The band at 1304 cm^-1^ found in the FTIR spectrum of myofibers [6, 12] but absent in the spectra of ECM is a specific signature in the amide III region of myofibers. As shown in Figure 4A, 4B and 4C, there were no differences in the FTIR spectra of ECM structural proteins or myofibers between the RV and LV of control pigs. Accordingly, the myofiber/collagen indicator 1 [A(1171 cm^-1^)/A(1338 cm^-1^)] associated with the myofiber plasma membrane (Figure 5A) and the myofiber/collagen indicator 2 [A(1304 cm^-1^)/A(1338 cm^-1^)] associated with the myofiber specific components (Figure 5B), collagen I mRNA (Figure 5C), collagen III mRNA (Figure 5D) and collagen III protein (Figure 5E), were identical in the RV and LV in control pigs. Immunohistochemistry images showed the lack of fibrosis in the control ventricles (Figure 3).

**FTIR pattern of protein secondary structures in control myocardium**

As an averaging technique, FTIR spectroscopy simultaneously provides information concerning all proteins present in the tissue, and it is thus not possible to attribute specific amide absorptions to peculiar secondary structures within individual proteins. Curve fitting of the spectra was performed using Gaussian-Lorentzian functions for each absorption band, which were fixed from the minima of the second derivative spectra, and secondary structures were expressed as the percentage of total amide I absorption. As shown in **Figure S2A**, the averaged second derivative spectra present different minima in the amide I/amide II zone, suggesting a contribution of different types of secondary conformations of proteins tissues [2] such as α helices, β sheets and turns, random conformations, etc. The position and therefore the assignment of these different minima is the same in the four series of samples. The amounts of these different protein conformations can be extracted from the decomposition of the FTIR spectra in the amide I zone and they been reported in **Figure S2B**. As shown in **Figure S2B**, these amounts were rather preserved in dilated samples. We only observed a slight increase in band 1632 cm^-1^ in dilated ventricles, assigned to hydrogen bonded carbonyl groups in intramolecular β sheets structures.

**Myocardial FTIR lipid pattern (cholesteryl ester, free cholesterol, triglycerides, fatty acids and phospholipids) in control pigs**

The complex FTIR spectra of pig ventricles also include lipids with their classic markers in the 2800-3000 cm^-1^ region (especially the CH_2_ stretching of long hydrocarbon chains) (Figure 4A) and in the 1475-1450 cm^-1^ region (CH_2_ scissoring and CH_3_ bending) (Figure 4B). These lipids mainly include the phospholipids of plasmatic membranes (confirmed by the presence of the C=O stretching of ester groups and the CO-O-C and PO_2_^-^ stretching bands (Table S1), as well as triglycerides, cholesteryl esters, free cholesterol and free fatty acids (C=O stretching at 1712 cm^-1^ and COO^-^ stretching at 1392 cm^-1^). Unsaturated lipids possess a specific marker at 3013 cm^-1^.

**Myocardial HPTLC phospholipid pattern in control and DCM pigs**

Myocardial pig samples contain the following phospholipid species: L-α-phosphatidylcholine (PC), L-α-phosphatidylethanolamine (PE), sphingomyelin (SM) and cardiolipin (CL), with PC being the most abundant (Figure S3). There were no differences in PC, PE, SM and CL contents between the ventricles of control pigs.

| Band position (cm^-1^) | Assignment |
| --- | --- |
| 3283 | Amide A  Mainly the ν(N-H) mode of proteins with the contribution of the ν(O-H) stretching mode of H_2_O and polysaccharides |
| 3072 | ν(C-H) aromatic |
| 3013 | ν(C=H) of unsaturated lipids, triglycerides, fatty acids |
| 2957, 2923, 2871, 2852 | νas(CH_3_), νas(CH_2_), νs(CH_3_),νs(CH_2_),  (Gly, Pro, Hyp, and Ala) of proteins + phospholipids, triglycerides  Most representative of proteins: νs(CH_3_)  Most representative of lipids: νas(CH_2_) and νs(CH_2_) |
| 1740 | ν(C=O) of the ester carbonyl groups of phospholipids and triglycerides |
| 1712 | ν(C=O) of the nucleoside side of nucleic acids and ν(C=O) of free fatty acids |
| 1646 | Amide I ν(C=O)  Multi-component band sensitive to protein secondary structure  Better resolved in second derivative or FSD spectra |
| 1540 | Amide II ν(C-N), δ (N-H)  Multi-component band sensitive to protein secondary structure  Better resolved in second derivative or FSD spectra |
| 1515 | Tyrosine band |
| 1471, 1452 | δ(CH_2_) scissoring, δ(CH_3_) bending of lipids (mainly), proteins |
| 1392 | νs(COO-) of free amino acids, fatty acids, δ(CH_3_) |
| 1338 | δ(CH_2_) wagging of the proline chain  ***Specific band of collagen*** |
| 1320-1200 | Amide III δ_plan_ (N-H) and ν(C-N) of proteins  Sensitive to secondary structure  ***1235 cm^-1^: specific to the ECM***  ***1204 cm^-1^ : specific to the ECM (collagen)***  ***1304 cm^-1^: specific to myofibers (creatin)*** |
| 1246-1235 | νas(PO_2_^-^) stretching of phospholipids, nucleic acids |
| 1226 | ν(SO_4_^2-^) of proteoglycans |
| 1200-1000 | ν(C-O), ν(C-C), ν(C-OH), ν(C-O-C) of proteins, oligosaccharides, glycolipids and νs(PO_2_^-^) nucleic acids, phospholipids  Better resolved in second derivative or FSD spectra  **In particular**  1171-1154 cm^-1^: νas(CO-O-C) of cholesterol esters, phospholipids  1171 cm^-1^ νas(CO-O-C) ***specific to myofibers***  1159 cm^-1^ : ν(C-OH) of Hyp and nucleic acids  1120 cm^-1^ : ν(C-O) lactate, polysaccharides, glycogen (***specific to myofibers)***  1089-1100 cm^-1^ : νs(PO_2_^-^)  1079 cm^-1^: ν(C-O-C) collagen, glycogen, oligosaccharides, glycolipids, and proteoglycans (***specific to the ECM)***  1042 cm^-1^ : νs(CO-O-C) of carbohydrates residues and polysaccharides |
| 972 | Phosphorylated proteins, phospholipids, and nucleic acids |
| 929 | υ(Cα-C)characteristic of α helices |

**Table S1: Absorption bands of control pig ventricles**

**Figure S1.** Comparison of cardiomyocyte size between porcine and human right and left ventricles of control samples. **A**) Haematoxylin/eosin staining of pig and human right and left control ventricles. **B**) Bar graphs showing the quantification of cardiomyocyte length and width in right and left ventricles. Pigs: N=5; Human: N=3. Results are expressed as mean ± SD. ***P*<0.01 *vs* pig samples; ****P*<0.005 *vs* pig samples.

**Figure S2.** Minor alterations in protein secondary structures in pig dilated ventricles. **A**) Averaged second derivative spectra of pig ventricles in the Amide I/II zone **B**) Bar graphs showing the quantification of the main secondary structures, N=5/group. Results are expressed as mean ± SD.

**Figure S3**. Phospholipid content of right and left ventricles in control and dilated pigs. Representative thin layer chromatography (TLC) (**A**) and bar graphs showing the content of L-α-Phosphatidylcholine (PC) (**B**), L-α-Phosphatidylethanolamine (PE) (**C**), Sphingomyelin (SM) (**D**) and Cardiolipin (**E**), N=7/group Results are expressed as mean ± SD. RV: right ventricle; LV: left ventricle.

**Figure S4**. Hydric composition of right and left ventricles of control and DCM pigs. Bar graphs show total (A), freezable (B) and unfreezable (C) water composition of right and left ventricles in control and dilated pigs, N=5/group. Results are expressed as mean ± SD. RV: right ventricle; LV: left ventricle.

**REFERENCES**

[1] **Aktas N, Tülek Y, Gökalp HY**. Determination of freezable water content of beef semimembranous muscle DSC study. *J. Therm. Anal.* 1997; 48; 259–66.

[2] **Staniszewska E, Malek K, Baranska M**. Rapid approach to analyze biochemical variation in rat organs by ATR FTIR spectroscopy. *Spectrochim. Acta. A. Mol. Biomol. Spectrosc.* 2014; 118; 981–6.

[3] **Wang Q, Sanad W, Miller LM, et al.** Infrared imaging of compositional changes in inflammatory cardiomyopathy. *Vib. Spectrosc.* 2005; 38; 217–22.

[4] **Gough KM, Zelinski D, Wiens R, et al.** Fourier transform infrared evaluation of microscopic scarring in the cardiomyopathic heart: effect of chronic AT1 suppression. *Anal. Biochem.* 2003; 316; 232–42.

[5] **Zohdi V, Wood BR, Pearson JT, et al.** Evidence of altered biochemical composition in the hearts of adult intrauterine growth-restricted rats. *Eur. J. Nutr.* 2013; 52; 749–58.

[6] **Kirschner C, Ofstad R, Skarpeid H-J, et al.** Monitoring of denaturation processes in aged beef loin by Fourier transform infrared microspectroscopy. *J. Agric. Food Chem.* 2004; 52; 3920–9.

[7] **Bozkurt O, Severcan M, Severcan F**. Diabetes induces compositional, structural and functional alterations on rat skeletal soleus muscle revealed by FTIR spectroscopy: a comparative study with EDL muscle. *Analyst* 2010; 135; 3110.

[8] **Petibois C, Gouspillou G, Wehbe K, et al.** Analysis of type I and IV collagens by FT-IR spectroscopy and imaging for a molecular investigation of skeletal muscle connective tissue. *Anal. Bioanal. Chem.* 2006; 386; 1961–6.

[9] **Cheheltani R, Rosano JM, Wang B, et al.** Fourier transform infrared spectroscopic imaging of cardiac tissue to detect collagen deposition after myocardial infarction. *J. Biomed. Opt.* 2012; 17; 56014.

[10] **Belbachir K, Noreen R, Gouspillou G, et al.** Collagen types analysis and differentiation by FTIR spectroscopy. *Anal. Bioanal. Chem.* 2009; 395; 829–37.

[11] **Bromberg PS, Gough KM, Dixon IM**. Collagen remodeling in the extracellular matrix of the cardiomyopathic Syrian hamster heart as assessed by FTIR attenuated total reflectance spectroscopy. *Can. J. Chem.* 1999; 77; 1843–55.

[12] **Jerônimo DP, de Souza RA, da Silva FF, et al.** Detection of creatine in rat muscle by FTIR spectroscopy. *Ann. Biomed. Eng.* 2012; 40; 2069–77.
